# Role of Intestinal Microbiota in Baicalin-Induced Drug Interaction and Its Pharmacokinetics

**DOI:** 10.3390/molecules21030337

**Published:** 2016-03-10

**Authors:** Keumhan Noh, Youra Kang, Mahesh Raj Nepal, Ki Sun Jeong, Do Gyeong Oh, Mi Jeong Kang, Sangkyu Lee, Wonku Kang, Hye Gwang Jeong, Tae Cheon Jeong

**Affiliations:** 1 College of Pharmacy, Yeungnam University, Gyeongsan 38541, Korea; nkhj007@ynu.ac.kr (K.N.); kyr27@ynu.ac.kr (Y.K.); maheshnpl10@gmail.com (M.R.N.); balunboy@naver.com (K.S.J.); cucucu10@hotmail.com (D.G.O.); mjkang@ynu.ac.kr (M.J.K.); 2 College of Pharmacy, Kyungpook National University, Daegu 41566, Korea; sangkyu@knu.ac.kr; 3 College of Pharmacy, Chung-Ang University, Seoul 06974, Korea; wkang@cau.ac.kr; 4 College of Pharmacy, Chungnam National University, Daejeon 34134, Korea; hgjeong@cnu.ac.kr

**Keywords:** baicalin, baicalein, drug interaction, pharmacokinetics, intestinal microbiota

## Abstract

Since many glycoside compounds in natural products are hydrolyzed by intestinal microbiota when administered orally, it is of interest to know whether their pharmacological effects are derived from the glycoside itself or from the aglycone form *in vivo*. An interesting example is baicalin *versus* baicalein, the aglycone of baicalin, which is contained in some herbs from Labiatae including *Scutellaria baicalensis* Georgi and *Scutellaria lateriflora* Linne. The herbs have been extensively used for treatment of inflammatory diseases in Asia. Although there have been numerous reports regarding the pharmacological effects of baicalin and baicalein *in vivo* and *in vitro*, some reports indicated that the glycoside form would hardly be absorbed in the intestine and that it should be hydrolyzed to baicalein in advance for absorption. Therefore, the role of metabolism by intestinal microbiota should also be considered in the metabolism of baicalin. In addition, baicalin contains a glucuronide moiety in its structure, by which baicalin and baicalein show complex pharmacokinetic behaviors, due to the interconversion between them by phase II enzymes in the body. Recently, concerns about drug interaction with baicalin and/or baicalein have been raised, because of the co-administration of *Scutellaria* species with certain drugs. Herein, we reviewed the role of intestinal microbiota in pharmacokinetic characteristics of baicalin and baicalein, with regards to their pharmacological and toxicological effects.

## 1. Introduction

More than 400 bacterial species have been identified in the human feces, and most of them are anaerobic bacteria [1]. The amounts and species of intestinal bacteria are different across the location of the gastrointestinal (GI) tract, and the amounts of bacteria are increased exponentially from the stomach to colon. In general, the number of bacterial cells in the stomach and duodenum are reportedly 10^1^ and 10^3^ cells/g, respectively, and those in colon are about 10^11^–10^12^ cells/g [2,3]. The predominant species of intestinal bacteria is also dependent on the site of the intestine [4]. Whereas the bacteria which belong to *Bacillus* subgroup of Firmicutes and Actinobacteria are predominant in the small intestine, Bacteriodetes and the Lachnospiraceae family of Firmicutes are more prevalent in the colon [3,4].

The intestinal microbiota play a critical role in not only human health and disease but also the pharmacokinetics of xenobiotics, such as drugs and toxicants, because various enzymes (e.g., β-glucuronidase, sulfatase and various glycosidases) produced by intestinal microbiota could affect the absorption and metabolism of these compounds in the intestine [1,5,6,7,8]. The consequences of xenobiotic metabolism by intestinal microbiota would be increased toxicity by the production of toxic or carcinogenic metabolites, decreased toxicity or detoxication, delayed excretion of xenobiotics by enterohepatic circulation, production of pharmacologically active metabolites, species variation in metabolism and toxicity, differences in efficacy and toxicity resulting from the routes of administration, and production of metabolites not formed in tissues, which were summarized in the literature elsewhere [6,8]. In particular, glycosidic compounds are strongly affected by enzymes produced by intestinal microbiota, so that the change of intestinal microbiota can lead to changes in pharmacokinetics, pharmacodynamics and toxicity of glycosidic compounds [6,9,10,11,12]. In this regard, most of the isolated compounds having glycosidic linkages from natural products and herbs can be easily hydrolyzed to the corresponding aglycones by enzymes in the intestine [6]. In addition, numerous studies to date have been done only *in vitro*, which would make the experimental results difficult to interpret. Therefore, it is of interest to know whether the parent glycosidic compounds, their aglycone forms, or both forms have pharmacological and toxicological effects *in vivo*.

**Figure 1:**
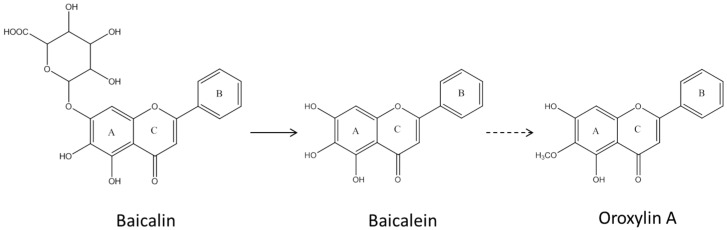
Proposed pathway of baicalin metabolism by human intestinal microbiota [15]. The solid and dashed lines represent major and minor pathways, respectively.

Baicalin (baicalein 7-*O*-glucuronide, Figure 1) and baicalein, the aglycone form, are naturally occurring compounds from *Scutellaria baicalensis* Georgi, and the roots of this herb have been used as a traditional herbal medicine in East Asia [13]. The various bioactivities and pharmacological effects of baicalin and baicalein, such as anti-inflammation, anti-cancer, and anti-pruritic effects, have been reported in the literature [14,15,16]. Baicalin is metabolized to baicalein by β-glucuronidase in the intestine [12], and this metabolic process is a critical stage for absorption of baicalin [6,12]. Of equal importance is that baicalin contains a glucuronic acid moiety rather than glucose. Because of the glucuronic acid that is also used in the phase 2 metabolism of glucuronidation in body, baicalin can be formed in tissues, even though it is metabolized to baicalein, the aglycone form, for absorption in the intestine by microorganisms. In fact, once baicalein is absorbed in the intestine, baicalin, baicalein 6-*O*-glucuronide, and baicalein 6,7-diglucuronide are detected in the blood [12].

Herein, we reviewed the effects of intestinal microbiota on pharmacokinetics, pharmacodynamics, and toxicity of baicalin and baicalein. The pharmacokinetic interactions with certain drugs induced by either baicalin or baicalein were also summarized in the present study.

## 2. Role of Intestinal Microbiota in Pharmacokinetics of Baicalin and Baicalein

### 2.1. Pharmacokinetic Change of Baicalin and Baicalein by Intestinal Microbiota

Orally administered baicalin is easily metabolized to baicalein by β-glucuronidase produced by the intestinal microbiota like *E. coli*, and the enzyme activity expressed by *E. coli* in the GI tract is as strong as that originated from either bovine or rat liver [17,18,19]. The produced baicalein is well absorbed from the intestine into the body, and most of baicalein absorbed is reconverted to baicalin and baicalein 6-*O*-glucuronide by UDP-glucuronosyltransferase in either small intestine or liver. This first-pass effect is responsible for low bioavailability of xenobiotics including baicalein following single oral administration [20,21]. In addition, it has been well accepted that baicalein would be the major form that is absorbed in the intestine, but not baicalin [20]. The literature indicated that the kinds and numbers of intestinal microbiota in individuals might affect the pre-systemic metabolism and absorption process of baicalin in the intestine, and that those factors would finally lead to a significant pharmacokinetic change of baicalin.

To demonstrate the effect of intestinal microbiota on the pharmacokinetics of certain compounds, antibiotic-treated animals and germ-free animals have been widely employed [6,7,8,22]. Likewise, the pharmacokinetic changes of baicalin and baicalein have also been tested in these animal models. For an example, Kang and colleagues compared pharmacokinetic parameters of baicalin between conventional and antibiotic-treated rats [12]. In the study, the rats were pretreated with either saline or a mixture of antibiotics (*i.e.*, cefadroxil, oxytetracycline, and erythromycin) for three days, and 100 mg/kg of baicalin was orally administered two days after the last dose of antibiotics in rats. As a result, the total numbers of bacteria in small intestine, cecum, and large intestine were reduced by about 81.5%, 60.0%, and 64.3%, respectively, in antibiotic-pretreated rats, and the maximum serum concentration (C_max_) and the area under the serum concentration-time curve (AUC) for baicalin in antibiotic-pretreated rats were also reduced by 66.5% and 49.0%, respectively [12].

Xing *et al.* [23] evaluated the absolute bioavailabilities of baicalin and baicalein in both normal and antibiotic-pretreated rats. Two kinds of antibiotics, neomycin and streptomycin, were orally given for six days, and baicalin was administered to rats by an oral administration at 224 μmol/kg. The results showed that the calculated absolute bioavailabilities based on the AUC of the parent baicalin form in normal and antibiotic-pretreated rats were 2.2% and 1.5%, respectively [23]. However, the results also showed that those values for baicalin obtained from the AUC of the total baicalein after enzymatic hydrolysis using β-glucuronidase/sulfatase in normal and antibiotic-pretreated rats were 28.0% and 7.7%, respectively, and that significantly reduced bioavailability of baicalin was obtained in antibiotic-pretreated rats when compared with normal rats. Meanwhile, the absolute bioavailability of baicalein based on the AUC of the total baicalein in antibiotic-pretreated rats following an oral administration with 224 μmol/kg was not markedly changed when compared with normal rats [23]. The results clearly indicated that antibiotics would decrease in the absorption of baicalin by reducing the hydrolysis process in the GI tract without affecting the absorption process of baicalein, and that metabolism of baicalin to baicalein by the intestinal microbiota would be a very critical step for the absorption of baicalein [23].

The role of intestinal microbiota in pharmacokinetics of baicalin was confirmed again in germ-free rats. Germ-free animals are animals that would not have any intestinal microbiota, which would be an ultimate animal model for investigating the role of intestinal microbiota in the metabolism of certain drugs in the intestine. After an oral administration with baicalin to conventional and germ-free rats, baicalin could be detectable in plasma prepared from the conventional rats even 24 h after the administration [24]. However, only a small amount of baicalin was detected in the plasma samples of germ-free rats even 2 h after the administration, at which the Cmax is achieved in conventional animals. Therefore, the obtained AUC of baicalin in germ-free rats was 12.0% of that obtained in conventional rats. The recovered amounts of baicalin and baicalein from GI tract 4 h after an oral administration with baicalin were 13.4% and 21.9% of administered dose in conventional rats, respectively. In contrast, a substantial proportion of baicalin (55.1%), but only a trace amount of baicalein, was observed in the GI tract of germ-free rats [24].

Taken all together, it could be concluded that baicalin might require metabolism to baicalein by intestinal microbiota for absorption, and that the absorbed baicalein could be transformed to baicalin and other glucuronidated forms by UDP-glucuronosyltransferase in the body.

### 2.2. Effects of Various Factors Affecting the Growth of Intestinal Microbiota on the Pharmacokinetics of Baicalin and Baicalein

The pattern of intestinal microbiota present in the intestine, which can affect the pharmacokinetics of baicalin and baicalein, can be altered by various factors. A disease, such as obesity or type 2 diabetes, is one of the most important factors contributing to the composition of intestinal microbiota [25].

When fresh feces obtained from type 2 diabetic mice were incubated with Radix Scutellariae extract for 48 h in anaerobic conditions, the produced amounts of baicalein were remarkably increased in comparison with those from normal mice, and oroxylin A, a methylated baicalein, was only found in the samples isolated from type 2 diabetic mice [26]. Based on this finding, Xu *et al*. [26] proposed different metabolic pathways of baicalin in normal and type 2 diabetic mice (Figure 1).

Deng and colleagues also reported the differences in baicalin metabolism in type 2 diabetic rats *in vitro* and *in vivo* when compared with normal rats [27]. After the incubation of Radix Scutellariae extract in fecal suspensions and intestinal mucosa homogenates for 1 h, respectively, the produced amounts of baicalein in type 2 diabetic rats were 1.4-fold higher than those in normal rats. Furthermore, the activity of intestinal β-glucuronidase in type 2 diabetic rats was significantly increased by 2.3-fold [27]. The changes in enzyme activity could induce the pharmacokinetic change of baicalin because the hydrolysis of baicalin in the intestine could affect the pre-systemic metabolism and absorption process of baicalin. When 1.19 g/kg Radix Scutellariae extract was administered to normal and type 2 diabetic rats, the AUC_∞_ of baicalin in type 2 diabetic rats was significantly increased by about 2.2-fold in comparison with that in normal rats, which was consistent with the increase in β-glucuronidase activity [27].

In contrast, no pharmacokinetic changes of baicalin were observed in allergy-induced rats [28]. After an oral administration with 1667 mg/kg KOB extract which is a polyherbal medicine for the treatment of hyperhidrosis, containing 100 mg/kg baicalin, to normal and allergy-induced rats, the obtained pharmacokinetic parameters, such as C_max_ and AUC of baicalin, in allergy-induced rats were similar with those in normal rats [28].

The demographic factors, such as age and sex, can also cause the change of intestinal microbiota. Yim *et al*. [29] evaluated the metabolic activity of baicalin with its metabolites by human fecal specimens obtained from persons of any age of either sex. The results showed that the metabolic activity of baicalin to baicalein was highly variable regardless of age and sex. So, inter-individual variability of the metabolic activity in the intestine must be closely related with differences in the production of enzymes metabolically active for baicalin to baicalein, rather than with differences of species or amounts of intestinal microbiota [19,30]. Although the species and amounts of intestinal microbiota were not extensively studied in the studies, many literatures recently reported indicated significant changes in intestinal microbiota by factors of age and sex [31,32].

## 3. Role of Intestinal Microbiota in Efficacy and Toxicity of Baicalin and Baicalein

### 3.1. Anti-Cancer Effects of Baicalin and Baicalein

The anti-cancer effect of baicalin and baicalein has been demonstrated through many studies, and these compounds showed comparable inhibitory effects on the growth of various cancer cell lines based on the IC_50_ values [18,19]. However, some studies indicated that baicalin and baicalein have different anti-cancer activities in several cancer cell lines including prostate and bladder cancer cells [33,34]. Chen *et al*. [33] reported that the IC_50_ values for baicalin and baicalein in an androgen-positive human prostate cancer cell line, LNCaP, were 60.8 and 29.8 μM, respectively, and those in an androgen-negative human prostate cancer cell line, JCA-1, were 46.8 and 17.7 μM, respectively. In contrast to the prostate cancer cells, Ikemoto *et al*. [34] evaluated dose-dependent anti-proliferative activities on human bladder cancer cell lines, KU-1 and EJ-1, and a murine bladder cancer cell line, MBT-2. The results indicated that baicalin might have much stronger anti-proliferative effects on KU-1 and EJ-1 cell lines than baicalein. In addition, comparable inhibitory effects were obtained by baicalin and baicalein in the cell proliferation of MBT-2. Nevertheless, the effect of intestinal microbiota on the anti-cancer effect of baicalin and baicalein has not been evaluated *in vitro* and *in vivo*. When considering the pharmacokinetic characteristics of baicalin, which is hydrolyzed to baicalein in the intestine and regenerated in the intestine/liver after absorption [35], it is easily speculated that the change in intestinal microbiota might also induce the change in anti-cancer effect of baicalin and baicalein. In this regard, baicalein was hardly detected in rats when baicalin was orally treated, due to the rapid conversion to baicalin and other glucuronidated metabolites by phase 2 enzymes once absorbed, indicating the clinical relevance of baicalin in anticancer activity [12]. Therefore, further studies are needed to evaluate the impact of intestinal microbiota on anti-cancer effects induced by baicalin and baicalein *in vitro* and/or *in vivo* in the near future.

### 3.2. Anti-Inflammatory Effect of Baicalin and Baicalein

Jung *et al*. [36] evaluated the change in anti-inflammatory effect of baicalin by intestinal microbiota in mice treated with antibiotics. The mice either with or without antibiotics pre-treatment were given a single oral dose of 20 mg/kg baicalin, and lipopolysaccharide (LPS) was intraperitoneally injected to mice 6 h after vehicle or baicalin administration. Then, the levels of pro-inflammatory cytokines, such as tumor necrosis factor (TNF)-α, interleukin (IL)-1β and IL-6, in the serum were determined 4 h after the LPS injection. The results indicated that treatment with baicalin significantly decreased in the levels of pro-inflammatory cytokines in both normal and antibiotic-treated mice [36]. However, antibiotic treatment significantly attenuated the anti-inflammatory effect of orally administered baicalin in mice [36]. Furthermore, when LPS was given to normal mice pretreated with baicalin or its metabolites (*i.e.*, baicalein or oroxylin A) intraperitoneally, the serum levels of TNF-α, IL-1β and IL-6 were only significantly reduced in mice pretreated with either baicalein or oroxylin A with the most potent inhibition by oroxylin A [36]. The results suggested that the intestinal microbiota might play a critical role in the anti-inflammatory effect of baicalin by metabolizing baicalin to baicalein and oroxylin A in the intestine.

### 3.3. Anti-Pruritic Effect of Baicalin and Baicalein

The role of intestinal microbiota in the anti-pruritic effect of baicalin was explored in animal models by Trinh and colleagues [16]. When either baicalin, baicalein, or oroxylin A was orally administered either 1 or 5 h before treatment with scratching agents, such as either histamine or compound 48/80, in mice, the maximum anti-scratching behavioral effect by baicalin was observed in mice pretreated 5 h before injection with scratching agent. However, the administration with baicalin metabolites (*i.e.*, baicalein or oroxylin A) 1 h before injection with scratching agents showed more potent inhibitory effects on the scratching behavior [16]. Furthermore, the anti-scratching behavioral effect induced by baicalein or oroxylin A was more potent after intraperitoneal administration than after oral administration, and was comparable in mice with or without antibiotics. In contrast, the inhibitory effect of baicalin on histamine-induced scratching behavior was significantly reduced in mice treated with antibiotics compared to normal mice. The results indicated that, to generate the anti-scratching behavioral effect of baicalin, it might require a time of at least 6 h for baicalin to be metabolized to baicalein and/or oroxylin A, and that these metabolites produced by intestinal microbiota are responsible for the anti-scratching behavioral effect of baicalin [16]. Although the Tmax of baicalin following an oral administration was reportedly 4 h in rats [12], the pharmacokinetic profile in oroxylin A production was not presented in the study [16].

### 3.4. Toxicity of Baicalin and Baicalein in HepG2 Cells

From a toxicological viewpoint, baicalin would be more toxic than baicalein. Therefore, the intestinal microbiota have a protective effect against hepatotoxicity induced by baicalin. Khanal *et al*. [37] investigated cytotoxicity and apoptotic effects of baicalin and baicalein in HepG2 cells. The results indicated that baicalin was more cytotoxic than baicalein in HepG2 cells, and the number of apoptotic cells and DNA fragmentation by baicalin were also much higher than baicalein. When baicalin was incubated with human fecal suspension (fecalase), however, the cytotoxic effect in HepG2 cells was abolished in a concentration-dependent manner, and apoptotic cell death was also significantly decreased [37]. These results clearly indicated that metabolism of baicalin by human fecalase to baicalein might have a protective effect against baicalin-induced toxicity in HepG2 cells [37].

## 4. Baicalin and Baicalein Induced *in Vivo* Drug Interaction

### 4.1. Metabolic Enzymes and Plasma Protein Binding Displacement-Mediated Drug Interaction

Baicalin- or baicalein-induced pharmacokinetic interactions with certain drugs were summarized in Table 1. However, to date, all the studies were done without consideration of the possible role of intestinal microbiota. As mentioned earlier, much evidence indicates that metabolism of baicalin to baicalein and other metabolites might be significantly affected by intestinal microbiota [12,16,19,20,21], and that baicalin and baicalein could interact with some CYPs and transporter proteins as presented in Table 1. Because some significant interaction would be possible, investigating the role of intestinal microbiota in the studies outlined in the following in either antibiotic-pretreated or germ-free models should be given further consideration. After an intravenous injection of baicalin at 0.45 g/kg for investigating the interaction with phenacetin, a CYP1A2 substrate, to rats, several pharmacokinetic parameters of phenacetin, such as the plasma concentration at 60 min after drug administration (C_60min_), half-life (t_1/2_), volume of distribution (V_d_) and AUC were significantly increased, and the C_max_ and clearance (CL) were also increased by about 12% and 14%, respectively, when compared with control group [38]. Furthermore, the percentage changes of control in pharmacokinetic parameters of phenacetin (C_60min_, t_1/2_, V_d_, and AUC) were correlated with the C_max_ of baicalin, and the unbound fraction of phenacetin also increased in a plasma baicalin concentration-dependent manner. It suggested that the inhibitory effect of baicalin on CYP1A2 activity and increase in unbound phenacetin by baicalin might contribute to the pharmacokinetic change of phenacetin following a simultaneous intravenous injection of phenacetin and baicalin [38]. The pharmacokinetics of theophylline was also affected by baicalin after the co-injection of those compounds intravenously. Those changes in C_max_, t_1/2_, CL and AUC of theophylline by baicalin may be also attributed to two mechanisms, *i.e.*, plasma protein binding displacement and inhibition of CYP1A2 activity [39].

Meanwhile, Noh *et al*. [40] reported that, although baicalin inhibited CYP1A activity in rat liver microsomes *in vitro*, there was no difference between pharmacokinetics of caffeine and its metabolites (*i.e.*, paraxanthine, theobromine, and theophylline), when caffeine was orally administered 8 h after single intragastric administration with baicalin (0.2 g/kg) in rats, because sufficient plasma concentration of baicalin for CYP inhibition could not be reached.

After a single intravenous injection of midazolam, a representative CYP3A substrate, with baicalin (0.225, 0.45, and 0.9 g/kg) in rats, CL of midazolam was decreased by about 25%–34% in a dose dependent manner, and AUC_∞_ was significantly increased by 47%–53% when compared to control group [41]. Similar pharmacokinetic change of midazolam was also shown after repeated intravenous administration of 0.9 g/kg baicalin for a week. Following repeated doses of baicalin, the CL and AUC_∞_ of midazolam were decreased by 43% and increased by 53%, respectively. These results were mainly due to the competitive inhibition of baicalin on CYP3A-mediated midazolam metabolism in the rat liver [41].

Cheng *et al*. [42] evaluated the pharmacokinetic change of nifedipine after intravenous co-administration with nifedipine and baicalin (0.225 or 0.45 g/kg) in rats. V_d_ and CL of nifedipine in rats treated with low dose of baicalin were increased 1.85- and 1.97-fold more than those in control rats, respectively, and those in rats treated with high doses of baicalin were also increased 3.24- and 3.42-fold more when compared to control group, respectively. The C_max_ and AUC_∞_ of nifedipine were significantly decreased by 40% and 41% in rats with low dose of baicalin, and 65% and 63% in rats with high dose of baicalin, respectively. The calculated CLs in those groups were increased by 97% and 242%, respectively [42]. *In vitro* results demonstrated that baicalin not only was a competitive displacer of nifedipine, but also inhibited CYP3A activity in rat liver microsomes in a concentration-dependent manner. Taken together, it was concluded that the pharmacokinetic changes of nifedipine in rats treated with baicalin might be caused by inhibitory effects of baicalin on plasma protein binding and CYP3A-mediated metabolism [42].

Gao and colleagues conducted the pharmacokinetic study of chlorzoxazone, a CYP2E1 probe drug, in rats either with or without baicalin co-administration based on the same study design mentioned above [42,43]. When chlorzoxazone was intravenously injected with low (0.225 g/kg) or high (0.45 g/kg) doses of baicalin, in rats, t_1/2_ of chlorzoxazone was prolonged by 34% in the low dose group and by 53% in the high dose group. Furthermore, V_d_ of chlorzoxazone in low- and high-dose groups were significantly increased by 37% and 50%, respectively, and C_max_ was decreased by 25% in the low dose group and 33% in the high dose group. Meanwhile, AUC and CL of chlorzoxazone were comparable between rats both with and without baicalin [43]. The reduction of plasma protein binding and inhibition of CYP2E1 activity by baicalin might be responsible for the pharmacokinetic changes of chlorzoxazone *in vivo* [43].

### 4.2. Transporter-Mediated Drug Interaction

Baicalin and baicalein can affect the pharmacokinetics of certain drugs by regulating various transporters involved in the drug absorption, distribution, metabolism and excretion [44]. Again, unfortunately, the possible role of intestinal microbiota has not been studied.

Fan *et al*. [45] evaluated the pharmacokinetic change of rosuvastatin, a substrate of organic anion-transporting polypeptide 1B1 (OATP1B1), after the co-administration of rosuvastatin and baicalin tablets (three times daily orally for two weeks) in healthy subjects who were CYP2C9*1/*1 with different OATP1B1 haplotypes (OATP1B1*1b/*1b, OATP1B1*1b/*15, and OATP1B1*15/*15). Among three different haplotypes of *OATP1B1*, the t_1/2_, AUC_72hr_ and AUC_∞_ of rosuvastatin were significantly decreased by 16.2%, 47.0% and 41.9% in OATP1B1*1b/*1b, and 18.0%, 21.0% and 23.9% in OATP1B1*1b/*15, respectively. The obtained CL in OATP1B1*1b/*1b and OATP1B1*1b/*15 were increased by 173.5% and 130.7%, respectively, when compared to control (placebo) group for each haplotype. In contrast, there was no pharmacokinetic change of rosuvastatin by baicalin in OATP1B1*15/*15 [45]. These results indicated that baicalin reduces plasma concentration of rosuvastatin in an *OATP1B1* haplotype-dependent manner, and that induction of hepatic rosuvastatin uptake via *OATP1B1* might partially contribute to this phenomenon [45].

Baicalein is known as a p-glycoprotein (P-gp) inhibitor, and the planar structure of baicalein is important for the interaction with P-gp [46]. The inhibitory effect of baicalein on P-gp has been demonstrated through *in vivo* studies. When nimodipine, one of the P-gp substrates, was orally administered 0.5 h after a single oral administration of baicalein (0.4, 2 or 8 mg/kg) in rats, the AUC_∞_ of nimodipine in rats treated with 2 and 8 mg/kg baicalein were increased by 1.4- and 1.6-fold, respectively [47]. The obtained C_max_ of nimodipine was also significantly increased in the presence of baicalein (2 and 8 mg/kg). In contrast, there was no pharmacokinetic change of nimodipine following intravenous co-administration of nimodipine and baicalein in rats. In the same study, *in vitro* baicalein inhibited CYP3A4 activity and enhanced the cellular uptake of rhodamine-123, a P-gp substrate, in a concentration-dependent manner. It indicated that the pharmacokinetic change of nimodipine by baicalein might be due to inhibition of CYP3A4-mediated metabolism and/or P-gp efflux of nimodipine in the small intestine and liver [47].

After administration of cyclosporine, which is a substrate of both P-gp and CYP3A4, with baicalin (112 μmol/kg) in rats, C_max_ and AUC_540min_ of cyclosporine were significantly increased by 408.1% and 685.3% after treatment with baicalin, respectively [48]. In rats with co-administration of baicalein (112 μmol/kg), those pharmacokinetic parameters were also elevated by 87.5% and 150.2%, respectively. However, there was no difference in the elimination phase of cyclosporine, regardless of treatment with either baicalin or baicalein.

Li *et al*. [49] demonstrated the pharmacokinetic interaction of baicalein with tamoxifen, which is a substrate of P-gp and CYP3A4, in rats. After a single oral administration with baicalein (0.5, 3, and 10 mg/kg), AUC_∞_ and C_max_ of orally administered tamoxifen in the presence of baicalein were significantly increased by 47.6%–89.1% and 54.8%–100.0%, respectively. The CL of tamoxifen in rats treated with either 3 or 10 mg/kg baicalein were reduced by 34.3% and 48.6%, respectively. Furthermore, the metabolite-parent AUC ratio of tamoxifen after treatment with 3 and 10 mg/kg baicalein was also significantly decreased. It indicated that the metabolism of tamoxifen by CYP3A4 was affected by baicalein [49].

Although extensive studies on the possible role of intestinal microbiota in drug interaction induced by either baicalin or baicalein have not been investigated, the results presented above would suggest further investigations, because the production and appearance of baicalin and baicalein in tissues following absorption can be greatly influenced by the intestinal microbial condition. In this regard, a recent report that only baicalein, but not baicalin, could inhibit the P-gp activity indicated that the possible change in the distribution of baicalin and baicalein by intestinal condition would change the systemic exposure to certain drugs [50].

## 5. Conclusions and Future Directions

As reviewed, the absorption process of baicalin and baicalein is strongly affected by intestinal microbiota, and one of the most powerful factors that induces a change in the intestinal environment, such as number and species of bacteria, would be antibiotics. The global consumption of antibiotics increased by 36% between 2000 and 2010, and the Indians and Chinese were the largest and second largest consumers of antibiotics, respectively [51]. Furthermore, the high-income Asian countries and regions, such as Hong Kong, Malaysia, Singapore, and South Korea, ranked within the top eight markets of antibiotic consumption per person in 2010 [51]. When considering that *Scutellaria baicalensis* Georgi has been widely used in Asia, high rates of antibiotic use in Asia would reduce enzyme activities of intestinal microbiota in certain populations, and pharmacokinetics and pharmacodynamics of baicalin and baicalein may be altered by antibiotics. Therefore, more extensive and precise study is needed to evaluate the effects of intestinal microbiota, not only on pharmacokinetic/pharmacodynamics changes of baicalin and baicalein, but also drug interaction between either baicalin or baicalein and certain drugs *in vivo*. So, we suggest the following approaches for the safe and effective use of baicalin and baicalein.

First, further investigation on the pharmacokinetic change of baicalin and baicalein by intestinal microbiota is needed. So far, most *in vivo* studies have focused on the pharmacokinetics of baicalin and its aglycone, baicalein, after administration. However, a substantial amount of administered baicalin was metabolized to oroxylin A, methylated baicalein, and its conjugates in various organs [52]. Furthermore, mice treated with oroxylin A showed more potent inhibitory effects on the scratching behavior and inflammation than mice treated with baicalin [16,36], and oroxylin A was only observed after baicalin incubation with intestinal microbiota of type 2 diabetic mice [26]. Taken together, the metabolic alteration of baicalin by intestinal microbiota in the intestine should be investigated in more detail *in vivo*. Likewise, the identification of intestinal microbiota involved in the metabolism of either baicalin or baicalein would be necessary in the near future with the individual variability. Information on individual variability in intestinal microbiota would predict individual differences in the efficacy and toxicity of baicalin and baicalein. Although the specific types of intestinal microbiota metabolizing baicalin to baicalein have not been extensively investigated, it was well documented that a variety of antibiotic treatments that reduce the intestinal microbiota-mediated metabolism of drugs and natural products could influence systemic exposure to them [53].

Secondly, precise characterization of pharmacological and toxicological changes of baicalin and baicalein by intestinal microbiota is required. So far, although most studies have focused on the pharmacokinetic alteration of these compounds by intestinal microbiota, the effect by these pharmacokinetic changes has not been widely studied *in vivo*. For instance, although anti-cancer effect of baicalin and baicalein has been demonstrated through *in vitro* and *in vivo* studies [33,34,54], the influence of intestinal microbiota on anti-cancer effects of those compounds has not been elucidated. Particularly, a little is known about toxicological alteration of baicalin by intestinal microbiota change *in vivo*. Khanal *et al*. [37] reported that intestinal microbiota reduced baicalin-induced toxicity in HepG2 cell, but it has not been demonstrated through *in vivo* studies. So, this approach would provide an understanding of the role of intestinal microbiota in pharmacological and toxicological actions of baicalin and baicalein.

Finally, the effect of intestinal microbiota on pharmacokinetic interaction of either baicalin or baicalein with certain drugs should be clearly elucidated *in vivo*. Although lots of studies have demonstrated the pharmacokinetic change of certain drugs by either baicalin or baicalein, there has been no study that explained the possible role of intestinal microbiota in baicalin-induced pharmacokinetic changes of certain drugs. As mentioned above, the change in intestinal microbiota can alter the pharmacokinetics of baicalin and baicalein, which might affect the pharmacokinetics of co-administered drugs. For the evaluation of effects of intestinal microbiota, the administration route of either baicalin or baicalein and test drugs in clinical settings should be carefully considered. In this regard, some studies which evaluated the drug interaction of either baicalin or baicalein with certain drugs have been conducted following the intravenous co-injection of these compounds *in vivo* [38,39,42,43]. When considering the pre-systemic metabolism of baicalin and baicalein in the intestine, drug interaction study with either baicalin or baicalein should also be conducted after oral administration *in vivo*. In addition, human clinical trials would give the most valuable information on the role of intestinal microbiota in the pharmacokinetics of baicalin and/or the possible drug interaction of either baicalin or baicalein with certain drugs.

## Figures and Tables

**Table 1:** Summary of drug interactions with baicalin or baicalein *in vivo*.

Test Substances (Route of Administration)	Substrate Drugs (Route of Administration)	Pharmacokinetic Change of Substrate Drugs	Mechanism of Drug Interaction	Ref.
Baicalin (i.v.)	Phenacetin (i.v.)	C_max_ ↓, C_60min_ ↑, t_1/2_ ↑, V_d_ ↑, CL ↓, AUC_∞_ ↑	Plasma protein binding displacement CYP1A2 inhibition	[38]
Baicalin (i.v.)	Theophylline (i.v.)	C_max_ ↓, t_1/2_ ↑, V_d_ ↑, CL ↓, AUC_∞_ ↑	Plasma protein binding displacement CYP1A2 inhibition	[39]
Baicalin (p.o.)	Caffeine (p.o.)	No significant changes in parameters	Plasma baicalin was not enough for CYP inhibition	[40]
Baicalin (i.v.)	Midazolam (i.v.)	CL ↓, AUC_∞_ ↑	CYP3A inhibition	[41]
Baicalin (i.v.)	Nifedipine (i.v.)	C_max_ ↓, V_d_ ↑, CL ↑, AUC_∞_ ↓	Plasma protein binding displacement CYP3A inhibition	[42]
Baicalin (i.v.)	Chlorzoxazone (i.v.)	C_max_ ↓, t_1/2_ ↑, V_d_ ↑	Plasma protein binding displacement CYP2E1 inhibition	[43]
Baicalin (p.o.)	Rosuvastatin (p.o.)	t_1/2_ ↓, CL ↑, AUC_∞_ ↓	OATP1B1 induction	[45]
Baicalein (p.o.)	Nimodipine (p.o.)	C_max_ ↑, AUC ↑	CYP3A inhibition P-gp inhibition	[47]
Baicalin (p.o.)	Cyclosporine (p.o.)	C_max_ ↑, AUC ↑	CYP3A inhibition P-gp inhibition	[48]
Baicalein (p.o.)	Cyclosporine (p.o.)	-	CYP3A inhibition P-gp inhibition	[48]
Baicalein (p.o.)	Tamoxifen (p.o.)	C_max_ ↑, AUC ↑, CL ↓	CYP3A inhibition P-gp inhibition	[49]
